# Suppression of PMA-induced tumor cell invasion and migration by ginsenoside Rg1 via the inhibition of NF-κB-dependent MMP-9 expression

**DOI:** 10.3892/or.2014.3422

**Published:** 2014-08-20

**Authors:** LI LI, YIWEN WANG, BENQUAN QI, DONGDONG YUAN, SHUYING DONG, DAOHUA GUO, CUILING ZHANG, MEILING YU

**Affiliations:** 1 Department of Pharmacy, The First Affiliated Hospital of Bengbu Medical College, Bengbu, Anhui 233004, P.R. China; 2 Faculty of Pharmacy, Bengbu Medical College, Bengbu, Anhui 233030, P.R. China; 3 Department of Pharmacy, Sun Yat-Sen Memorial Hospital, Sun Yat-Sen University, Guangzhou, Guangdong 510120, P.R. China; 4 Department of Emergency Internal Medicine, The First Affiliated Hospital of Bengbu Medical College, Bengbu, Anhui 233004, P.R. China; 5 Department of Anesthesiology, The Third Affiliated Hospital of Sun Yat-Sen University, Guangzhou, Guangdong 510700, P.R. China

**Keywords:** ginsenoside Rg1, MMP-9, NF-κB, metastasis

## Abstract

Ginseng has become one of the most commonly used alternative herbal medicines, and its active component, ginsenoside Rg1 has known pharmacological effects, including anticancer properties. However, the effects of ginsenoside Rg1 on metastasis have yet to be investigated. In this study, we demonstrated the ability of ginsenoside Rg1 to suppress phorbol myristate acetate (PMA)-induced invasion and migration in MCF-7 breast cancer cells. MCF-7 cells were treated with ginsenoside Rg1 and incubated with or without PMA. The protein and mRNA expression of MMP-9 and MMP-2 was analyzed using Transwell and wound-healing assays and western blotting. The results showed that suppression was associated with the reduced secretion of MMP-9, a key metastatic enzyme. MMP-9 levels were regulated transcriptionally and correlated with the suppression of NF-κB phosphorylation and DNA binding activity. Conversely, ginsenoside Rg1 did not affect MMP-2 mRNA and TIMP-1 mRNA levels, or the activation of AP-1, suggesting a specificity of pathway inhibition. Inhibition of NF-κB activation by p65 small-interfering RNA (siRNA) was shown to suppress PMA-induced cell invasion and migration. The siRNA studies also showed that PMA-induced MMP-9 expression is NF-κB-dependent. The results suggested that the anticancer properties of ginsenoside Rg1 may derive from its ability to inhibit invasion and migration, and that these processes are regulated in breast cancer cells through the NF-κB-mediated regulation of MMP-9 expression.

## Introduction

The invasion and metastasis of cancer cells are known to be primary causes of cancer progression (1). When tumor cells metastasize, a number of proteolytic enzymes contribute to the degradation of ECM components and the basement membrane (2,3). Metalloproteinases (MMPs) play an essential role in the process and promotion of tumor invasion and metastasis in many types of cancer (4).

Among the previously reported MMPs, MMP-9 and MMP-2 are key enzymes for degrading ECM and type IV collagen (5,6). MMP-9 correlates with malignant phenotypes in various types of cancer and can be activated by a variety of stimuli such as cytokines and phorbol myristate acetate (PMA) during varied pathological processes (7,8). The MMP-9 promoter region contains one NF-κB and two AP-1 binding sites (9–11), which are absent in the MMP-2 promoter region. Therefore, the activation of MMP-9 in cancer progression may be derived in part from its modulation by AP-1 and NF-κB transcription factors in response to extracellular stimuli.

Ginseng has become one of the most commonly used alternative herbal medicines, and ginsenoside Rg1 is one of its most active and abundant components. Ginsenoside Rg1 has pharmacological effects in the central nervous, cardiovascular, and immune systems, and also exerts anticancer properties (12–20). However, the effect of ginsenoside Rg1 on cancer metastasis remains to be investigated. In the present study, we demonstrated the effects of ginsenoside Rg1 on PMA-induced invasion and migration in breast cancer and examine the potential mechanism involved in these effects. Our results support a model by which ginsenoside Rg1 inhibits MMP-9 expression by suppressing NF-κB activation to inhibit PMA-induced invasion and migration in MCF-7 cells.

## Materials and methods

### Materials

Ginsenoside Rg1 was purchased from Shanghai Yaji (Group) Co., Ltd. (Shanghai, China). Sulforhodamine B (SRB), trichloroacetic acid (TCA), acetic acid, anti-β-actin and dimethyl sulfoxide (DMSO) were obtained from Sigma-Aldrich (St. Louis, MO, USA). TRIzol and cell culture reagents were purchased from Invitrogen Life Technologies, (Carlsbad, CA, USA). Antibodies for phospho-p65, phospho-c-jun and MMP-9 were obtained from Cell Signaling. Secondary antibodies for western blotting were obtained from Amersham Biosciences Corporation (Piscataway, NJ, USA). Other reagents were obtained from Sigma-Aldrich unless stated otherwise.

### Sulforhodamine B (SRB) assay

Cytotoxicity was determined by the SRB assay. Cells were seeded into 96-well plates and exposed to different concentrations (50, 100, 200 and 400 μM) of ginsenoside Rg1. After 48 h of incubation, the cells were fixed with TCA for 1 h at 4°C, air-dried, and then stained with 0.4% SRB solution for 30 min at room temperature. After staining, the SRB solution was removed, and the cells were subsequently washed five times with 1% acetic acid. Then, 10 mM Tris base solution (pH 10.5) was added to dissolve the protein-bound dye, and plates were incubated on a plate shaker for 10 min. The OD570 nm was determined using a 96-well plate reader (MRX; Dynex Technologies, Chantilly, VA, USA).

### Western blotting

MCF-7 cells were seeded in 6-well plates and exposed to the indicated concentrations of ginsenoside Rg1 with or without PMA. After treatment, the cells were harvested using lysis buffer (pH 7.4, 20 mM Tris-HCl, 150 mM NaCl, 1 mM EDTA, 1 mM EGTA, 1% Triton, 2.5 mM sodium pyrophosphate, 1 mM Na_3_VO_4_, 1 mM β-glycerophosphate, 1:1,000 protease inhibitors). Protein concentrations were determined by the BCA method. Total protein (25 μg) was then separated using 8–12% sodium dodecyl sulfate-polyacrylamide gels and transferred to nitrocellulose blotting membranes. The membranes were probed with monoclonal antibodies against MMP-9, MMP-2, TIMP-1 or β-actin (1:8,000), and immunopositive bands were visualized using the Amersham ECL™ Plus Western Blotting Detection kit (GE Healthcare, Piscataway, NJ, USA).

### Electrophoretic mobility shift assay (EMSA) (21,22)

Biotin 3′ end-labeled DNA probes containing the NF-κB consensus site: sense 3′-TCAACTCCCCTGAAAGGGTCCG-5′ and antisense 5′-AGTTGAGGGGACTTTCCCAGGC-3′, were purchased from Invitrogen (Shanghai, China). EMSA was performed using the light shift chemiluminescent EMSA kit (Pierce, Rockford, IL, USA). Briefly, the nuclear proteins were incubated in 1X binding buffer, 50 ng/μl poly (dI•dC), 0.05% NP-40, 5 mM MgCl_2_, 50 mM KCl, 2.5% glycerol and ddH_2_O for 20 min at room temperature in a total volume of 20 μl. The reaction mixture was separated on a 6% non-denaturing polyacrylamide gel and transferred to a positively charged nylon membrane. The membrane was cross-linked, and the biotin-labeled DNA was detected by chemiluminescence.

### Wound-healing assay

MCF-7 cells were seeded in a 6-well plate and incubated until they reached 80% confluence. A 200 μl pipette tip was used to create a wound, and the cells were washed twice with serum-free culture medium to remove floating cells and then replaced with fresh medium without serum. The cells were subjected to the indicated treatment for 24 h, and cells migrating from the leading edge were photographed at 0 and 24 h.

### Matrigel invasion assay

The invasion of MCF-7 cells was performed in a 24-well Transwell unit (8-μM pore size), which was coated with 1 mg/ml Matrigel matrix as previously described (23). Briefly, MCF-7 cells were placed on the Matrigel-coated Transwell (the upper compartment of the invasion chamber) in the presence or absence of Rg1 and PMA. Conditioned medium (500 μl) was added to the lower compartment of the invasion chamber. After incubation at 37°C for 48 h, the cells that had invaded the lower surface of the membrane were fixed with methanol and stained with hematoxylin and eosin. Random fields were counted by light microscopy.

### RNA extraction and reverse transcription polymerase chain reaction (RT-PCR)

TRIzol reagent was used to extract total RNA according to the manufacturer’s instructions. Complementary DNA (cDNA) was created from 1 μg RNA using standard procedures with avian myeloblastosis virus reverse transcriptase (Promega). For polymerase chain reaction (PCR) quantification, 2 μl of cDNA was amplified in a 20 μl standard PCR reaction. PCR was carried out by initial denaturation at 94°C for 3 min; 36 cycles of 94°C for 45 sec, 55°C for 45 sec, 72°C for 45 sec; and a final extension for 10 min at 72°C, followed by termination at 4°C. RT-PCR was performed using the following primer pairs (Invitrogen) for semi-quantitative assessment: sense 5′-TCCCTGGAGACC TGAGAA-3′ and antisense 5′-CGGCAAGTCTTCCGA GTAGTT-3′ for MMP-9; sense 5′-TGAGCTCCCGGAAAA GATTG-3′ and antisense 5′-TCA GCAGCCTAGCCAGTCG-3′ for MMP-2; sense 5′-GGGGCTTCA CCAAGACCTACAC-3′ and antisense 5′-AAGAAAGATGGGAGTGGGAACA-3′ for TIMP-1; sense 5′-CGTGGACATCCGCAAAGAC-3′ and anti-sense 5′-GCATTTGCGGTGGACGAT-3′ for β-actin. β-actin transcript served as an internal control for standardising the quantity of input cDNA. The PCR products were separated by electrophoresis on a 1.5% agarose gel and visualised under UV light using a gel documentation system (Bio-Rad, Hercules, CA, USA).

### Transient transfection and luciferase reporter assay

To determine promoter activity, MCF-7 cells were seeded in 6-well plates. At 70–80% confluence, the cells were cotransfected with pCMA-β-galactosidase plasmid along with pGL2-MMP-WT, pGL2-MMP-9-mAP-1–2 or pGL2-MMP-9-mNF-κB. The transfected cells were subsequently treated with ginsenoside Rg1 and stimulated with PMA. Following incubation for 2 h, the cells were lysed and luciferase activity was measured using a luminometer (Luminoscan Ascent; Thermo Electon Co., Germany).

### Zymography assay

Zymography was performed as previously described (23,24). Briefly, cells were incubated in serum-free RPMI-1640 and the supernatants were collected after incubation for 24 h. Conditioned media were collected, centrifuged and electrophoresed at 4°C on 10% SDS-PAGE containing 1 mg/ml gelatin. The gels were washed with 2.5% Triton X-100 and then incubated at 37°C for 24 h in a buffer containing 5 mM CaCl_2_, 50 mM Tris-HCl and 1 μM ZnCl_2_. The gels were stained with 0.05%Coomassie Brilliant Blue R-250 for 30 min at room temperature. The gelatinolytic activity of MMP-9 was observed as a white zone in a dark blue field.

### Transfection of siRNA

Human breast cancer MCF-7 cells were obtained from ATCC (Manassas, VA, USA) and seeded at 2×10^5^ cells/ml in 6-well plates. The cell plates were grown to 50% confluency and transfected with double-stranded siRNA for NF-κB p65 target sequence: (sense, 5′-CUUCCAAGUUCCUAUAGAAdTdT-3′ and antisense, 3′-dTdTGAAGGUU CAAGGAUAUCUU-5′) or with a siRNA non-specific control (Guangzhou RiboBio Co., Ltd., Guangzhou, China). Silencing was confirmed by western blotting and EMSA.

### Statistical analysis

Statistical analysis between groups was performed using an unpaired Student’s t-test with SigmaPlot 10.0 software (Jandel Scientific, San Rafael, CA, USA). Data are presented as means ± SEM. P<0.05 was considered to indicate a statistically significant result.

## Results

### Ginsenoside Rg1 inhibits cell invasion and migration of MCF-7 cells

**Figure 1:**
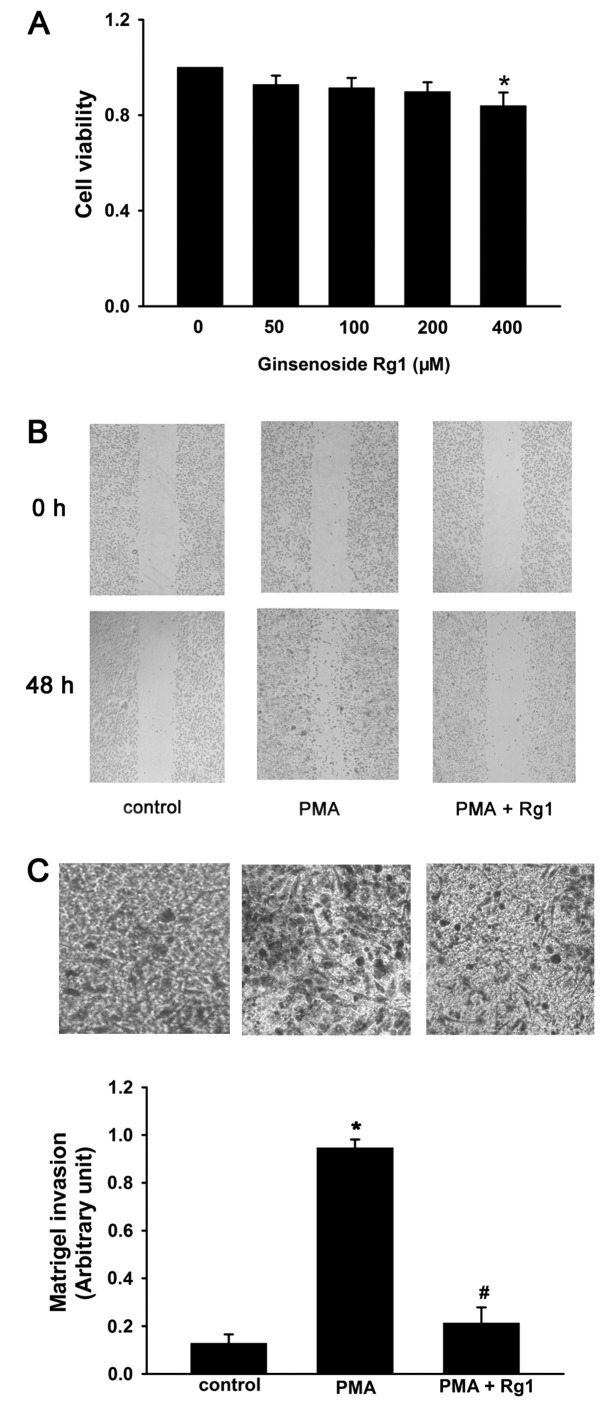
Ginsenoside Rg1 prevents the PMA-induced invasion and migration of MCF-7 cells. (A) Cells were treated with increasing concentrations of ginsenoside Rg1 (50–400 μM) for 48 h and the cell viability was assessed by the SRB assay. Results are the means ± SEM of three independent experiments normalized to 1 in the untreated sample; ^*^P<0.05 vs. untreated sample. (B) MCF-7 cells were scratched with a pipette tip, and the cells were exposed to 200 μM ginsenoside Rg1 for 48 h with or without 50 nM PMA for 24 h. Results are representative of three independent experiments. (C) The invasive ability of MCF-7 cells was assessed by a Matrigel invasion assay. The cells were incubated with 200 μM ginsenoside Rg1 for 48 h with or without 50 nM PMA for 24 h. Upper panel, representative images are shown; lower panel, quantification (means ± SEM) of three independent experiments normalized to 1 in the untreated sample; ^*^P<0.05 vs. control; ^#^P<0.05 vs. PMA group. PMA, phorbol myristate acetate.

To observe the effect of ginsenoside Rg1 on cell viability in MCF-7 cells, the cells were treated with increasing concentrations of ginsenoside Rg1 (50–400 μM) for 48 h and then assessed by SRB assay. Ginsenoside Rg1 had no effect on cell viability up to a concentration of 400 μM. Thus, the non-cytotoxic concentrations of ginsenoside Rg1, from 50 to 200 μM were used in subsequent experiments (Fig. 1A).

To investigate the effects of ginsenoside Rg1 on PMA-induced cell invasion and migration, Transwell and wound-healing assays were performed. The cells were pretreated with 50 nM PMA for 24 h and then exposed to ginsenoside Rg1 (50–200 μM) for 48 h. PMA significantly increased the invasion and migration of MCF-7 cells as compared with PMA-untreated control cells, while ginsenoside Rg1 reversed this effect (Fig. 1B and C). These results suggested that ginsenoside Rg1 may constitute an effective inhibitor of the invasion and migration in MCF-7 cells.

### Ginsenoside Rg1 suppresses MMP-9 secretion by inhibiting its protein and mRNA expression

**Figure 2:**
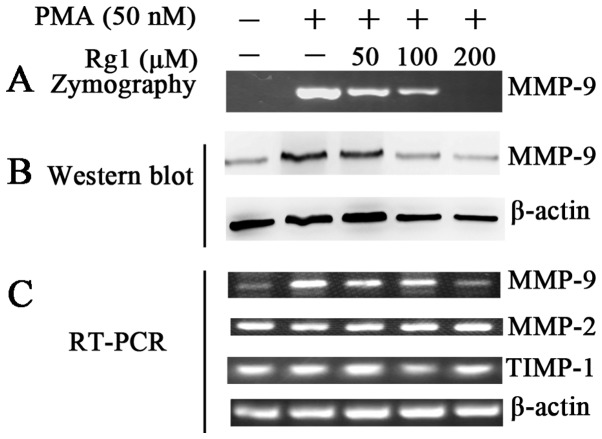
Ginsenoside Rg1 inhibits PMA-induced MMP-9 activity and expression. MCF-7 cells were treated with increasing concentrations (50–200 μM) of ginsenoside Rg1 (Rg1) for 48 h, followed by incubation with 50 nM PMA for 24 h. (A) MMP-9 activity in conditioned media was analyzed by zymography assay. (B) Cellular protein levels of MMP-9 were measured by western blotting. (C) The mRNA expression of MMP-9, MMP-2 and TIMP-1 was analyzed by RT-PCR. β-actin was tested as an internal control. Results are representative of at least three independent experiments. PMA group. PMA, phorbol myristate acetate.

MMP-9 and MMP-2 are important ECM-degrading enzymes that are involved in cancer invasion and metastasis (5–8). Zymography assay, western blotting and RT-PCR were used to determine the effects of ginsenoside Rg1 on PMA-induced MMP-9 activity, and the protein and mRNA expression (Fig. 2). The results showed that ginsenoside Rg1 suppressed PMA-induced MMP-9 activity in a dose-dependent manner, which may be explained by decreased levels of MMP-9 protein and mRNA. Ginsenoside Rg1 did not affect MMP-2 mRNA expression suggesting specificity for MMP-9. Since the activity of MMPs is regulated by the endogenous inhibitor TIMP-1, RT-PCR was used to determine the mRNA levels of TIMP-1. However, the mRNA levels of TIMP-1 were not significantly affected by ginsenoside Rg1 (Fig. 2C). This suggested that ginsenoside Rg1 suppressed PMA-induced MMP-9 secretion by affecting its transcription levels. However, the regulation of MMP-9 was not likely to occur at the level of regulation of TIMP-1 mRNA.

### Ginsenoside Rg1 inhibits PMA-induced MMP-9 activity by blocking its promoter activity

**Figure 3:**
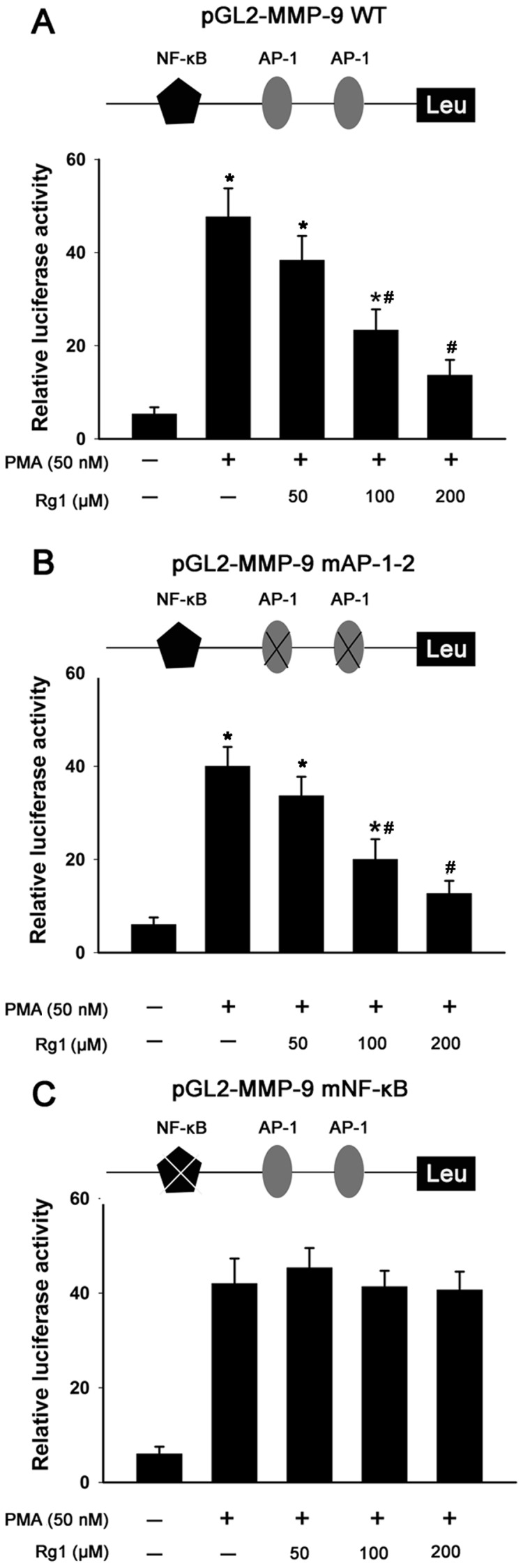
Ginsenoside Rg1 inhibits PMA-induced MMP-9 activity by blocking the transcriptional activity. MCF-7 cells were transfected with (A) pGL2-MMP-9wt or (B and C) pGL2-MMP-9-mAP-1–2 and pGL2-MMP-9-mNF-κB reporter plasmids containing mutations in the AP-1 and NF-κB binding sites, respectively. The cells were treated with ginsenoside Rg1 (50–200 μM) for 48 h, followed by incubation with 50 nM PMA for 24 h, and the luciferase activities in the cell extracts were assessed. Results (means ± SEM of triplicate wells) are representative of three independent experiments; ^*^P<0.05 vs. control; ^#^P<0.05 vs. PMA group. PMA, phorbol myristate acetate.

AP-1 and NF-κB transcriptional elements in the MMP-9 promoter are important in the regulation of its expression. To investigate the effect of ginsenoside Rg1 on PMA-induced MMP-9 promoter activity and the possible role of these elements, MCF-7 cells were transiently transfected with a wt-MMP-9 promoter luciferase reporter construct and reporter constructs with mutations in the AP-1 and NF-κB sites (MMP-9-mAP-1–2 or MMP-9-mNF-κB). The transcriptional activity of the wt-MMP-9 promoter reporter gene was activated up to 14-fold in cells by PMA. However, ginsenoside Rg1 significantly decreased the PMA-induced transcriptional activity of the reporter gene (Fig. 3A). Consistent with this result, the cells that received MMP-9-mAP-1–2 also demonstrated abrogated PMA-induced MMP-9 activity with ginsenoside Rg1 treatment (Fig. 3B). In contrast to the wt-MMP-9 and MMP-9-mAP-1–2, when cells were transfected with reporter plasmid containing the MMP-9-mNF-κB promoter, the luciferase activity of the cells induced by PMA was not affected by ginsenoside Rg1 (Fig. 3C). These results suggested that ginsenoside Rg1 inhibited PMA-induced MMP-9 activity by suppressing the transcriptional activity of NF-κB.

### Ginsenoside Rg1 inhibits NF-κB activation induced by PMA

**Figure 4:**
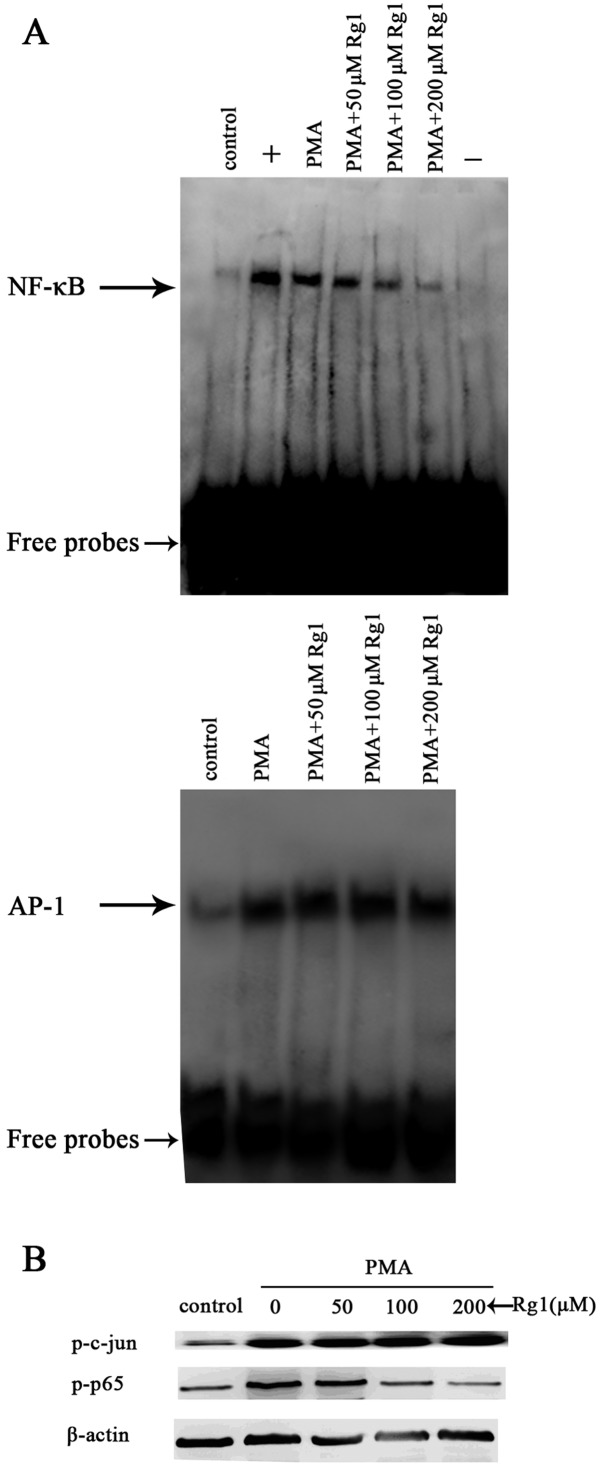
Ginsenoside Rg1 suppresses PMA-induced NF-κB activation. (A) MCF-7 cells were pretreated with ginsenoside Rg1 (50–200 μM) for 48 h, then exposed to 50 nM PMA for 2 h. Nuclear extracts were prepared and analyzed for NF-κB and AP-1 binding by EMSA. Lane ‘+’, positive-control (20 ng/ml TNF-α stimulation for 30 min); lane ‘−’, negative control (untreated cells). (B) Nuclear extracts were examined for phosphorylated levels of p65 and c-jun proteins by western blotting. β-actin levels were assessed as an internal control. Results are representative of three independent experiments. PMA, phorbol myristate acetate; EMSA, electrophoretic mobility shift assay.

To investigate the effect of ginsenoside Rg1 on PMA-induced NF-κB and AP-1 activity, we performed EMSA using binding elements from the MMP-9 promoter. MCF-7 cells were pretreated with ginsenoside Rg1 for 48 h and then exposed to 50 nM PMA for 2 h. PMA caused a modest, yet reproducible enhancement of the DNA binding activity of NF-κB, whereas ginsenoside Rg1 suppressed this activity in a dose-dependent manner (Fig. 4A, top panel). Conversely, although AP-1 was induced by PMA, ginsenoside Rg1 had no effect on PMA-induced AP-1 activity. These results suggested that ginsenoside Rg1 regulates the transcriptional activity of MMP-9 by inhibiting PMA-induced NF-κB, but not AP-1.

To confirm these results, the effect of ginsenoside Rg1 on the PMA-induced phosphorylation of p65, a major subunit of NF-κB; and c-jun, a major subunit of AP-1, were investigated by western blotting. Consistent with the EMSA data, the two proteins were phosphorylated in response to PMA. Furthermore, ginsenoside Rg1 suppressed the PMA-induced phosphorylation of p65 expression in a dose-dependent manner. However, it did not affect the phosphorylation of c-jun (Fig. 4B). These results are consistent with the possibility that NF-κB functions as a target of the inhibitory effects of ginsenoside Rg1 on MMP-9 expression.

### P65 suppresses siRNA cell invasion and migration of MCF-7 cells

**Figure 5:**
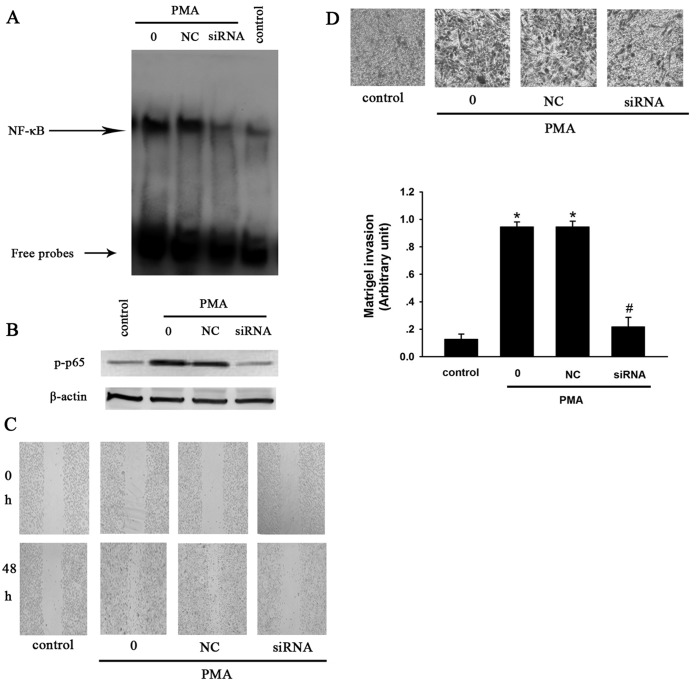
P65 siRNA inhibits PMA-induced cell invasion and migration. Cells were transfected with p65 siRNA (siRNA, 100 nM) or a negative control (NC, 100 nM) for 48 h, followed by treatment with 50 nM PMA for 2 h. (A) Nuclear extracts were analyzed for NF-κB activation by EMSA. (B) Western blot analysis was used to detect the effect of p65 siRNA on p-p65 expression. (C) MCF-7 cells were scratched with a pipette tip, and then cells were transfected with p65 siRNA or a negative-NC for 48 h, followed by treatment with 50 nM PMA for 24 h. Results are representative of three independent experiments. (D) The invasive ability of MCF-7 cells was assessed by a Matrigel invasion assay. Upper panel, representative images are shown; lower panel, quantification (means ±SEM) of three independent experiments normalized to 1 in the untreated sample; ^*^P<0.05 vs. control; ^#^P<0.05 vs. PMA group. PMA, phorbol myristate acetate; EMSA, electrophoretic mobility shift assay.

To confirm that PMA-induced MMP-9 expression is NF-κB-dependent, p65 siRNA was used to block the activation of NF-κB in the present study. MCF-7 cells were transfected with p65 siRNA for 48 h, followed by treatment with 50 nM PMA for 2 h. Western blotting and EMSA were used to investigate the expression of p-p65 and the activity of NF-κB. The results of the western blotting showed that PMA enhanced the expression of p-p65, while p65 siRNA reversed this effect. Consistent with the downregulation of p-p65 expression, NF-κB activation was shown by EMSA to be significantly inhibited by the siRNAs (Fig. 5A).

Transwell and wound-healing assays were subsequently performed to observe the effect of p65 siRNA on cell invasion and migration. The results demonstrated that PMA significantly promoted the invasion and migration of MCF-7 cells. However, p65 siRNA suppressed PMA-induced cell invasion and migration relative to the siRNA-negative controls (Fig. 5).

### P65 siRNA inhibits MMP-9 protein and mRNA expression by blocking its promoter activity

**Figure 6:**
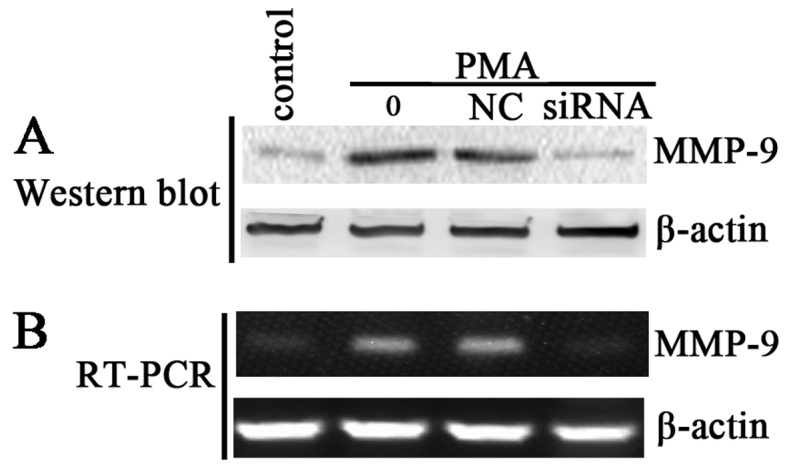
P65 siRNA inhibits PMA-induced MMP-9 protein and mRNA expression. Cells were transfected with p65 siRNA (siRNA, 100 nM) or a negative control (NC, 100 nM) for 48 h, followed by treatment with 50 nM PMA for 24 h. (A) Cellular protein levels of MMP-9 were measured by western blotting. (B) The mRNA expression of MMP-9 was analyzed by RT-PCR. β-actin was tested as an internal control. Results are representative of at least three independent experiments. PMA, phorbol myristate acetate.

**Figure 7:**
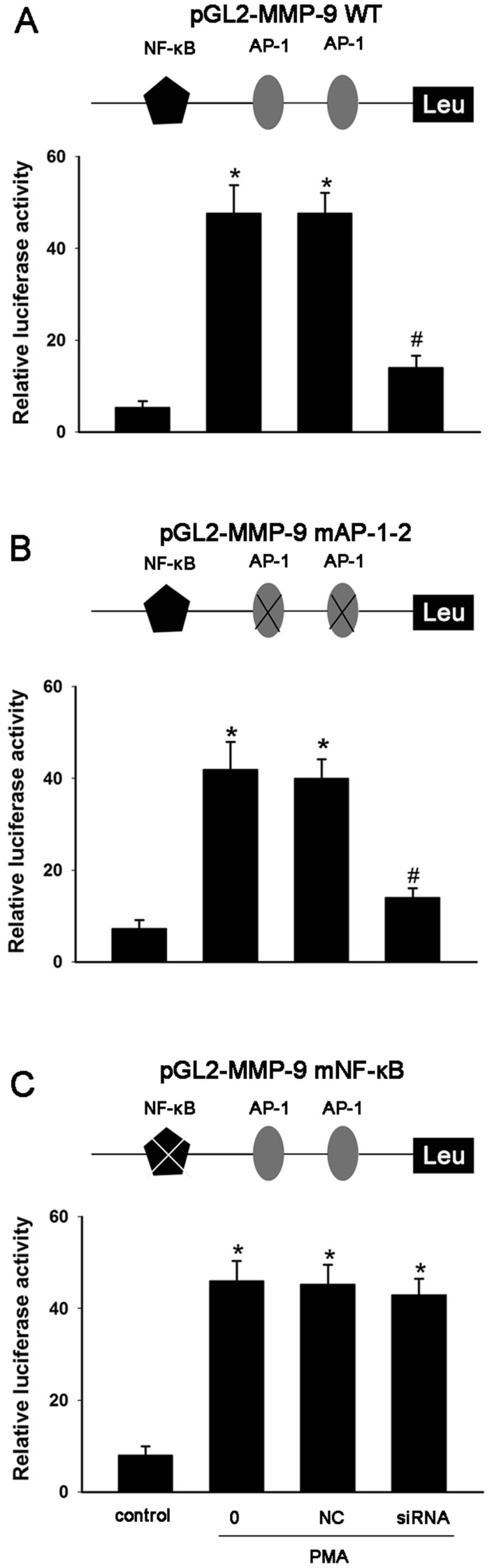
P65 siRNA inhibits PMA-induced MMP-9 activity by blocking the transcriptional activity. MCF-7 cells were transfected with (A) pGL2-MMP-9wt or (B and C) pGL2-MMP-9-mAP-1–2 and pGL2-MMP-9-mNF-κB reporter plasmids containing mutations in the AP-1 and NF-κB binding sites, respectively. Cells were transfected with p65 siRNA (siRNA, 100 nM) or an negative control (NC, 100 nM) for 48 h, followed by treatment with 50 nM PMA for 24 h, and the luciferase activities in the cell extracts were assessed. Results (means ± SEM of triplicate wells) are representative of three independent experiments; ^*^P<0.05 vs. control; ^#^P<0.05 vs. PMA group. PMA, phorbol myristate acetate.

The above results have demonstrated that ginsenoside Rg1 may suppress PMA-induced invasion and migration by inhibiting NF-κB-dependent MMP-9 expression. Thus, in the present study we detected the effect of p65 siRNA on the protein and mRNA expression of MMP-9. Results of western blotting and RT-PCR showed that p65 siRNA significantly inhibited the PMA-induced levels of MMP-9 protein and mRNA (Fig. 6). The effect of p65 siRNA on the promoter activity of MMP-9 was also investigated. MCF-7 cells were transiently transfected with a wt-MMP-9 promoter luciferase reporter construct and reporter constructs with mutations in the AP-1 and NF-κB sites (MMP-9-mAP-1–2 or MMP-9-mNF-κB). Consistent with the effect of ginsenoside Rg1, the results showed that p65 siRNA significantly inhibited PMA-induced MMP-9 activity by suppressing the transcriptional activity of NF-κB, but had no effect on the transcriptional activity of AP-1 (Fig. 7). Thus, the siRNA studies showed that PMA-induced MMP-9 expression is NF-κB-dependent. These results confirmed that ginsenoside Rg1 suppresses PMA-induced tumor cell invasion and migration by inhibiting NF-κB-dependent MMP-9 expression.

## Discussion

In the present study, we have demonstrated that ginsenoside Rg1 effectively suppresses PMA-induced invasion and migration. Ginsenoside Rg1 inhibits MMP-9 secretion by inhibiting its protein and mRNA expression, and since MMP-9 is known to act as a key regulator of metastatic function, it is likely that the suppressive effects of ginsenoside Rg1 on invasion and metastasis are mediated through its inhibition of MMP-9. Our results also show that ginsenoside Rg1 inhibits PMA-induced NF-κB activation in a dose-dependent manner, which may explain the transcriptional effects on MMP-9 mRNA. The siRNA studies show that PMA-induced MMP-9 expression is NF-κB-dependent. These results suggest that ginsenoside Rg1 serves as a potential inhibitor in preventing the invasion and metastasis of human breast cancers.

Invasion and metastasis are fundamental properties of cancer cells, and their control is therefore an important therapeutic goal. The identification of novel candidate agents that inhibit these processes is essential for preventing the progression of malignant tumors. In the present study, we investigated the effect of ginsenoside Rg1 on invasion and migration in MCF-7 breast cancer cells. PMA was used to induce cell invasion and migration based on its well-characterized role as a tumor promoter in chemical-induced carcinogenesis in many cancer cells, including hepatoma, colon, glioma and breast cancer cells (25–27). The Transwell and wound-healing assays demonstrated that PMA significantly induces invasion and migration in MCF-7 cells, while ginsenoside Rg1 suppresses these processes. These results suggest that ginsenoside Rg1 constitutes an effective inhibitor of cancer cell progression.

The enhanced expression of MMP-9 and/or MMP-2 has been shown to be responsible for PMA-induced cell invasion and migration (5–8). To investigate how ginsenoside Rg1 suppresses PMA-induced cell invasion and migration, we assessed the effects of ginsenoside Rg1 on PMA-induced MMP-9 by western blotting and RT-PCR. Ginsenoside Rg1 suppressed the PMA-induced MMP-9 protein and mRNA expression in a dose-dependent manner. However, ginsenoside Rg1 did not affect MMP-2 mRNA expression, suggesting a specificity of this response. The mRNA levels of TIMP-1 were also not affected by ginsenoside Rg1, suggesting that the suppression of PMA-induced MMP-9 is not mediated through TIMP-1.

However, one potential pathway of the regulation of MMP-9 is suggested by the results of the EMSA and western blot analyses. The results show that ginsenoside Rg1 suppresses PMA-induced NF-κB DNA-binding activity and the phosphorylation of p65, a major component of NF-κB that is known to translocate to the nucleus following phosphorylation and transactivate the promoters of multiple cancer-related genes, including MMP-9. Conversely, AP-1 DNA-binding activity and the PMA-induced phosphorylation of its c-jun subunit were not inhibited by ginsenoside Rg1, suggesting that the effects of ginsenoside Rg1 are limited to NF-κB. PMA is known to enhance the expression of MMP-9 through one NF-κB and two AP-1 binding sites within its promoter region (9–11). In the present study, the mutational analysis of the minimal MMP-9 promoter demonstrated a requirement for NF-κB, but not AP-1 for the suppression by ginsenoside Rg1. The siRNA studies also verified that PMA-induced MMP-9 expression is NF-κB-dependent. The above-mentioned results suggest a potential model whereby ginsenoside Rg1 inhibits PMA-induced MMP-9 activity through NF-κB to suppress breast cancer cell migration and invasion. Thus, ginsenoside Rg1 constitutes a potential antimetastatic and anti-invasive agent that may be useful in future clinical studies against cancer.
